# Dissertation on Practical Dentistry

**Published:** 1845-09

**Authors:** E. J. Dunning


### ARTICLE II.

*Dissertation on Practical Dentistry.* Read before the American Society of Dental Surgeons, at their Sixth Annual Meeting, held in the City of New York, August, 1845. By E. J. DUNNING.

HAVING been appointed by this society, at its last meeting, to read an essay on the present occasion, a sense of duty impels me to the performance of a task from which other considerations might cause me to shrink.

There are those around me whom I have the honor of greeting as brethren of the dental profession, whose years of experience, as operators, outnumber those of my whole life; whose youthful vigor, and maturer strength of mind and body, have been spent in unfolding the mysteries of dental disease, and discovering its remedies, in culling from the mechanic arts, the laboratory of the chemist, the cabinet of the mineralogist, and the furnace of the refiner, those sparks of knowledge which serve to light us on our toilsome way.

They have devoted years of study and experiment in their favorite science to bring it to its present degree of perfection, while their path has been constantly beset by obstacles, of which we can form no adequate conception. Surrounded by such as these, a young man should advance his opinions with great diffidence, and while detailing the results of his own experience, take the attitude of a scholar.

But these considerations ought not to deter any one, however young or inexperienced, from taking his part in our annual discussions, for in no other way can the important objects of our association be gained.

We meet for high purposes—to improve in knowledge, to add to the great fund of information which already exists, whatever of value we may have been able to glean from our year's experience.

The young and the old may join in this labor. Each brother, as he has passed from step to step in his professional career, has encountered obstacles which perhaps others have not; and has, by his own peculiar method of reasoning, arrived at a mode of surmounting them.

In this way, every member of the profession might, at our meetings, communicate the knowledge of some theory, manipulation, or remedy, which would be valuable to individuals, and honorable to the fraternity.

If every member should come to our conventions with his book of experience open wide, and plainly inscribed with each scrap of useful knowledge which his genius has gathered from the paths of practical science, how rich would be our yearly harvest! how gratifying and refreshing to leave the scenes of our toil, and join in the greetings of our cherished anniversary! If my humble mite shall, in the smallest degree, contribute to the interest of this occasion, my highest hopes with regard to it will be realized.

Before proceeding, however, to the discussion of the subjects which I have mainly proposed to myself in this essay, permit me to say a few words with reference to our conventions.

It would seem that the meetings of the members of a profession like our own, opening so broad a field for scientific investigation and practical improvement, should be characterised by efforts at mutual advancement in knowledge, that the time which we are permitted to spend in each other's society should be so improved that we may re-commence our labors with new courage, with deeper love for our profession and each other, and a stronger determination to prove a blessing to both.

Scattered throughout the length and breadth of our land, and engaged in the constant practice of our profession, are hundreds of those whose minds are constantly elaborating highly useful improvements. Men whose hearts are bound up in the love of their art, and the precious energies of whose minds are constantly devoted to its advancement. They are no misers! Their precious gleanings are free as air to their brethren and the world.

Visit the scenes of their toil—announce yourself as a professional brother, and your claim upon their hospitality is complete. The warm greeting, the generous grasp of the hand, and the glistening eye, pronounce you welcome—heartily welcome. They will lead you to their operating rooms and laboratories, and there open to you their caskets of jewels—processes which have cost them much time and labor to perfect—theories which have resulted from difficulties struggled with and overcome—apparatus which their ingenuity thus lays upon the shrine of their profession—they say it is all yours, it is all the world's!

Our society encloses within its pale many of these noble spirits; it professes for its object the advancement of our art—our mutual benefit and that of the world. Its meetings are announced, and with hearts burning with desire to accomplish this great object, they come from Maine and Louisiana, from the east and the west. Time and money are alike nobly sacrificed to be present at our discussions, and to give dignity and force to our association.

Why is it then that our sessions are so short, and so exclusively devoted to business transactions?

Why do we not spend a sufficient amount of time to allow every one who desires, or is willing to do so, to contribute his share to the interest and delight of the occasion?

In this way, and this only, may we retain the consideration and respect of the profession and the community, or even repay ourselves for the trouble of sustaining our organization.

How is it now? Our time is mostly employed in the election of officers and members, and the settlement of our financial and political affairs, while the great objects of our convention are neglected; and those even who have prepared themselves to instruct us cannot be allowed time to present the result of their labors.

In view of these things, who can wonder that more do not attend our meetings, when we offer a programme of such indifferent attractions. Does not rather the fact that so many from distant parts of the country are present, indicate an enthusiasm among our brethren worthy of a warmer reception.

I have taken occasion to present these suggestions, because I consider the subject to which they refer, of the highest importance to interests which we all hold dear. I leave them to the decision of your wisdom.

The remarks which I am about to offer will be practical, not confined to any particular department, but treating briefly various topics, to which my own attention is constantly directed.

It will not be surprising if they should contain no suggestions which are new or important to any one of you.

I can only say that I have chosen to describe a course of treatment which I have of late adopted in certain cases, and which I supposed might at least be novel to some.

I refer to those cases in which the nerve has become exposed by decay to the action of external agents.

Teeth affected in this manner are in many cases too valuable to the patient, both as regards their position in the arch, and their uses in mastication and articulation, to allow us to think for a moment of their extraction, until every means of rendering them comfortable and useful has failed.

Dentists have long been in the habit of operating upon these teeth with reference to their preservation, but it must be admitted that their efforts have been mostly confined to the incisors, while the grinders, considered less valuable, because less exposed, have been lost. Every effort to save even the more important dentals has been regarded as an experiment by some, and by others highly injudicious, while the grinders, frequently of the last importance to health and comfort, in their use of mastication, have been but too often lamentably sacrificed.

Viewed in this light, any useful suggestions, with reference to this most important department of dental surgery, are invested with the deepest interest. For if operations of this kind are ever successfully performed, they should undoubtedly rank among the noblest trophies of our art.

The measures which have been adopted in the treatment of exposed nerves have been various, and attended with different degrees of success. The great majority of authors whom I have consulted, as well as the practitioners with whose views I have become acquainted, recommend the reduction of its inflammation, and partial removal of sensibility, by the application of some powerful astringent preparation.

The use of the actual cautery is now, I believe, almost entirely abandoned, and those who have written within the last few years, generally recommend the continued application of galls, or the use of arsenious acid, combined with morphine.

The use of the latter preparation is now quite universal, there being but few practitioners who do not employ it to some extent. It acts with greater certainty, and accomplishes the object in less time than any other composition with which we are acquainted.

It however generally produces considerable pain for from three to twelve hours, and the inflammation thus kept up, frequently extends to the investing membrane and the gum, and results in suppuration of those parts.

Some have supposed that the use of arsenic under these circumstances might prove injurious to the general health through the action of the absorbents. It seems quite probable that such should be the case, when we consider the wonderful connection which exists throughout the whole system, and how mysteriously parts, apparently distant and disconnected, act upon each other.

Whether this be the case or not, its use by unskilful hands, and in immoderate quantities, is certainly attended with the danger of its reaching and acting directly upon the coats of the stomach.

My object, however, is not to enter into a discussion of the safety or utility of this application, but merely to describe the treatment which I have lately adopted in such cases.

The destruction of the nerve by mechanical means has been practised to a small extent by dental surgeons for many years. But on account of the severe pain which in many cases attends it, as well as the fact that in the manner in which it has generally been practised, it has proved no more successful than other and less severe methods, it has been considered rather in the light of a dernier resort.

To account for this, I think that it will be found that the nerve has been too frequently punctured merely, and then shut up by a stopping within its bony cell to decompose.

The putrescent and irritating matter thus generated, having no chance of escape, except at the end of the fang, must, of course, by its pressure at that point, induce pain, inflammation, and suppuration in the surrounding parts; the very common result of this method, as well as of the others which have been mentioned.

The only means of preventing this result is to remove the whole nerve, and fill its place with gold or tin foil.

This course, I, in common with others, have pursued for several years with reference to incisors and bicuspidati, but not until the past year have I adopted it to any great extent, with reference to molars.

It has been supposed that two very formidable obstacles would present themselves to the success of the operation upon this class of teeth.

1st. The excessive pain.

2d. The difficulty of removing the substance of the nerve from the fangs.

The first of these difficulties has been found much less than was anticipated; although it must be acknowledged that it varies much with the temperament of the individual, the general health of the mouth, and the degree of inflammation already existing in the exposed nerve.

There are many cases of highly nervous temperament combined with delicate health, in which it would undoubtedly be injudicious to attempt an operation, which, under such circumstances, must inevitably produce so great a shock upon the already enfeebled system. The health of the gums and teeth should also be considered. Every irritating cause should be removed before the operation is attempted.

If the gums are inflamed and spongy from the presence of tartar, or any other cause, or if the investing membrane is in a state of inflammation and partial suppuration, these diseases will constitute the strongest tendencies to the very difficulty which we would avoid, and render failure almost certain.

If the nerve should be found to be inflamed, as is frequently the case, owing to the presence in the cavity of irritating matter, the application of tannin, for some time before the operation, will render it much less painful.

This substance has also been found extremely useful in reducing inflammation in the bony structure, when the nerve is not exposed.

Although so much depends upon these circumstances, it will be found that judicious and skilful treatment will do much to remove this obstacle. In fact, it may be safely said, that in the majority of cases the pain of this operation will not be found to exceed that arising from the excavation of a sensitive cavity, or even the use of the file; at least such has been the testimony of many who have endured it; while others have declared that that which had been so frequently dreaded, as agony intolerable, was to them scarcely worthy of the name of pain.

The second difficulty, that of removing the nerve from the fang, is one which can be overcome only by perseverance and skill.

Every one who has mastered the art of stopping teeth, will remember how difficulties vanish before these potent charmers.

How the delicate manipulations, connected with this most beautiful operation, which weary and vex the clumsy fingers of the *beginner*, become comparatively easy and natural to the *adept*.

The instrument which I have used to excavate the fangs is a delicate probe of steel, perfectly annealed. The point may be converted into a very slight hook, and made sharp, so as to bring away the nerve or other matter with which the cavity may be filled. For the removal of the nerve in the chamber of the crown, existing in molar teeth, as well as to enlarge the cavity, so as to give free access to each of the fangs, a burr drill is very useful. As these teeth are generally very much decayed, it will be found advisable, when the cavity is on the side of the crown, to remove its edges in such a manner as to admit the light directly upon the openings of the fangs. This will facilitate the operation very much, and at the same time give strength to the walls that are to contain the stopping.

The operator, however, who has given himself every possible advantage, will frequently meet with mechanical difficulties in the way of the entire removal of the nerve, which will defy his skill. The external fangs of the upper molars, perhaps, will be those in which he will most frequently fail. Their internal cavities are frequently united in one broad, thin fissure, into which it would be perfectly impossible to introduce any instrument.

The quantity of nerve contained in these, is, of course, very small, compared with the whole; and as experience teaches us that this portion, although remaining in its place, alive, does not produce either pain or inflammation, we may consider it as of no serious importance. But this excavation should be carried as far as possible; for our motto, and the direction of all of our efforts, should be *perfection.*

Great care should be taken, in the course of this operation, to avoid passing the instrument through the opening at the end of the fang, which is frequently so large, especially in young persons, as to admit it. After having removed the nerve so far as practicable, the gold is prepared in very small pieces, which are immediately introduced, one by one, with a blunt probe, until each fang is thoroughly filled. The cavity in the crown is then stopped as usual.

So far as I have been able to observe the result, this operation has been successful in every case in which the nerve was destroyed, and its cavity filled at once. It has been tried in a large number of cases, and has never, to my knowledge, resulted in ulceration. But when the nerve had previously died, although it could not, as a general thing, remove disease already commenced, it has, in many cases, very sensibly diminished the frequency of its recurrence, and mitigated the violence of its symptoms.

In destroying the nerve in this manner, the pain occasioned is of such short duration, that it seems to produce no other effect upon the surrounding parts than a slight shock, the soreness resulting from which is usually felt for a day or two succeeding the operation.

Many of you have, undoubtedly, tried and decided upon the merits of this course. If this description shall prove interesting or of service to any, its intention will be most satisfactorily answered.

I propose to close with a few remarks upon the subject of stopping teeth. So much has already been said with regard to this most important operation, that it seems almost absurd for any one, especially a young man, to attempt to make any new suggestions with reference to it. Every one is aware of the necessity of removing every particle of carious matter; of giving such shape to the cavity, as is required to retain the gold; of freeing it from moisture; of introducing the foil in such a manner as to render every part compact, and perfectly adapted to the inequalities of the cavity; of giving it perfect solidity; and, although much has been said with reference to the finishing of the operation, still it may be that sufficient importance is not generally attached to it, in the minds of operators. All of the primary steps may be thoroughly and efficiently taken, but if the stopping is left in a rough and uneven state, the operation is imperfect. A slight indentation at the edge of the gold, where it joins the bone or enamel, may become a lodgment for acrid or decomposing matter, and the commencement of a new decay.

Now, although I do not believe that the greatest skill, exercised with consummate patience, can, in every case, be successful; still I do believe that there *may* be perfect operations, and that it should be the aim of every dentist to perform them.

The fact that decay is always directly induced by chemical agents, which are, of course, external, and which must find some roughness or indentation upon the enamel or bone, in which to lodge and remain for some time before they can produce the slightest effect, has been sufficiently proven by others. The very able paper from our brother Westcott, upon this subject, together with the investigation, a description of which accompanied it, are sufficiently convincing.

If this position is correct, the importance of giving not only a perfect surface to the stopping, but a surface perfectly continuous with that of the tooth commends itself to the consideration of every dentist whose aim is the highest excellence. In order to accomplish this, the first requisite is perfect solidity of the gold. Its surface should be made smooth with a fine file, which may be constructed of various shapes, to suit localities. The burnisher is then applied, which should be kept in good order, by rubbing upon a leather with crocus, and, to prevent the adhesion of the gold to its surface, should be touched to a piece of fine soap before using.

Having rendered the surface perfectly hard and solid in this manner, proceed to polish both the stopping and the tooth around it, especially if the bone or enamel has been filed. This may be done either with pumice stone and a stick of pine, or a pencil made of flour of emery and shellac, or a piece of any fine stone, as may best suit the case. Having removed all of the inequalities which might prove dangerous to the tooth, and especially those occurring just at the edge of the stopping, burnish the gold again, and you will have produced a fine surface, which will be retained, and which will many fold increase the probability of success.

In addition to this, impress upon your patient the necessity of perfect cleanliness—of removing, two or three times in each day, whatever may accumulate upon the teeth, and this not only upon the surfaces which may be washed by the brush, but between the teeth, where observation teaches us that decay is the earliest in its attacks, and the more dangerous, because concealed. In order to do this perfectly, the use of floss silk is necessary; I say *necessary*, because I know of nothing which will answer the same purpose, or even approach it in utility. The importance of this article cannot be over estimated; it should be kept and constantly recommended, and its use insisted upon, by every dentist. It protects the lateral surfaces of the teeth when in the closest contact, which cannot be reached or scarcely affected in the slightest manner by the brush. Will not reason then support us in the assertion that, in point of importance as a means of cleanliness, it is far above the brush, which, although of the greatest utility, affects only those parts which are constantly washed by the saliva, aided by the motions of the cheeks and tongue.

I have thus briefly, although imperfectly, performed the task assigned me. Full of errors though it be, it has been dictated by a desire *to do good.* I leave it with you, asking for its faults your generous forbearance.

